# A Latent Cardiomyocyte Regeneration Potential in Human Heart Disease

**DOI:** 10.1161/CIRCULATIONAHA.123.067156

**Published:** 2024-11-21

**Authors:** Wouter Derks, Julian Rode, Sofia Collin, Fabian Rost, Paula Heinke, Anjana Hariharan, Lauren Pickel, Irina Simonova, Enikő Lázár, Evan Graham, Ramadan Jashari, Michaela Andrä, Anders Jeppsson, Mehran Salehpour, Kanar Alkass, Henrik Druid, Christos P. Kyriakopoulos, Iosif Taleb, Thirupura S. Shankar, Craig H. Selzman, Hesham Sadek, Stefan Jovinge, Lutz Brusch, Jonas Frisén, Stavros Drakos, Olaf Bergmann

**Affiliations:** Centers for Regenerative Therapies Dresden (W.D., F.R., P.H., A.H., L.P., I.S., O.B.), Technische Universität Dresden, Germany.; Information Services and High-Performance Computing (J.R., F.R., L.B.), Technische Universität Dresden, Germany.; DRESDEN-Concept Genome Center, Technology Platform at the Center for Molecular and Cellular Bioengineering (F.R.), Technische Universität Dresden, Germany.; Departments of Cell and Molecular Biology (S.C., E.L., E.G., J.F., O.B.), Stockholm, Sweden.; Oncology-Pathology (K.A., H.D.), Karolinska Institute, Stockholm, Sweden.; National Board of Forensic Medicine (K.A., H.D.), Stockholm, Sweden.; European Homograft Bank, Brussels, Belgium (R.J.).; Department of Cardiothoracic and Vascular Surgery, Klinikum Klagenfurt and Section for Surgical Research Medical University Graz, Austria (M.A.).; Department of Cardiothoracic Surgery, Sahlgrenska University Hospital (A.J.), Gothenburg, Sweden.; Department of Molecular and Clinical Medicine, Institute of Medicine, Sahlgrenska Academy, University of Gothenburg (A.J.), Gothenburg, Sweden.; Department of Physics and Astronomy, Applied Nuclear Physics, Uppsala University, Uppsala, Sweden (M.S.).; Divisions of Cardiovascular Medicine and Cardiothoracic Surgery, University of Utah Health and School of Medicine (C.P.K., I.T., C.H.S., S.D.), Salt Lake City.; Nora Eccles Harrison Cardiovascular Research and Training Institute, University of Utah (C.P.K., I.T., T.S.S., C.H.S., S.D.), Salt Lake City.; The Sarver Heart Center and The Department of Internal Medicine/Cardiology, The University of Arizona College of Medicine Tucson, Arizona (H.S.).; Spectrum Health Frederik Meijer Heart and Vascular Institute and Van Andel Institute, Grand Rapids, MI (S.J.).; Department of Pharmacology and Toxicology, University Medical Center Goettingen (O.B.), Germany.; DZHK (German Centre for Cardiovascular Research), Lower Saxony Partner Site (O.B.), Germany.

**Keywords:** myocytes, cardiac, heart failure, heart-assist device ◼ polyploidy ◼ regeneration

## Abstract

**BACKGROUND:**

Cardiomyocytes in the adult human heart show a regenerative capacity, with an annual renewal rate of ≈0.5%. Whether this regenerative capacity of human cardiomyocytes is employed in heart failure has been controversial.

**METHODS:**

We determined cardiomyocyte renewal in 52 patients with advanced heart failure, 28 of whom received left ventricular assist device support. We measured the concentration of nuclear bomb test–derived ^14^C in cardiomyocyte genomic DNA and performed mathematical modeling to establish cardiomyocyte renewal in heart failure with and without LVAD unloading.

**RESULTS:**

We show that cardiomyocyte generation is minimal in end-stage heart failure patients at rates 18 to 50× lower compared with the healthy heart. However, patients receiving left ventricle support device therapy, who showed significant functional and structural cardiac improvement, had a >6-fold increase in cardiomyocyte renewal relative to the healthy heart.

**CONCLUSIONS:**

Our findings reveal a substantial cardiomyocyte regeneration potential in human heart disease, which could be exploited therapeutically.

**Clinical Perspective**

**What Is New?**

- Failing hearts show dramatically reduced cardiomyocyte renewal compared with healthy human hearts.
- Mechanical unloading with functional and structural improvement of the heart shows an increase in cardiomyocyte renewal compared with healthy hearts.

**What Are the Clinical Implications?**

- This study could redirect future translational investigations to identify new therapeutic targets for heart muscle regeneration in patients with chronic heart failure.

Loss of cardiomyocytes as a result of myocardial infarction or in the context of progressive cardiomyopathy can lead to heart failure, with considerable morbidity and mortality. Multiple strategies aiming to promote regeneration of the human myocardium are currently being explored. As cell replacement therapies using stem cell-derived cardiomyocytes still face considerable challenges,^1^ promotion of endogenous cardiomyocyte regeneration is an attractive alternative.

Cardiomyocytes in the healthy human heart have the capacity to renew for the lifetime of the individual, albeit at a low rate. In homeostasis, renewal occurs at a rate of ≈0.5% per year throughout adulthood, resulting in nearly 40% of the ventricular cardiomyocytes being exchanged during life.^2,3^ To what extent cardiomyocytes are regenerated in heart failure in humans is still poorly understood. Previous studies suggesting massive regeneration of cardiomyocytes proved difficult to reproduce,^4^ and identification of cardiomyocyte proliferation after a cardiac injury is challenging because of inflammation, scar formation, endoreplication, and the proliferation of other cell populations.^5,6^

Depending on the underlying disease, cardiomyopathies are categorized as ischemic cardiomyopathy (ICM) or nonischemic cardiomyopathy (NICM), with the latter comprising a heterogenous group of pathologies not primarily related to coronary artery disease. A left ventricular assist device (LVAD) is a pump that is surgically implanted in the left ventricle and helps propel blood, which is standard-of-care therapy for patients experiencing advanced heart failure and can be used as a bridge to heart transplantation or as a lifetime therapy. A subset of LVAD-supported patients attains significantly improved cardiac function and structure to the point at which withdrawal of the LVAD support can be considered.^7–11^ However, the underlying mechanisms for LVAD-mediated myocardial recovery are not fully understood,^12^ and it remains unknown whether new cardiomyocytes are generated in this process.^6,13,14^

In the present study, we retrospectively birth-dated cardiomyocytes from patients with cardiomyopathy by measuring ^14^C, derived from nuclear bomb tests during the Cold War, in genomic DNA, a method we developed to study cell renewal dynamics in humans^2,3,15^ (Figure S1). We established an integrated model of human cardiomyocyte turnover in cardiomyopathy that enables us to estimate renewal rates. Our data demonstrate that cardiomyocyte renewal is minimal in failing hearts but can elevate well beyond the levels observed in healthy hearts through LVAD-mediated functional cardiac improvement.

## METHODS

### Tissue Collection and Preparation

Heart tissue was procured from the European Homograft Bank (Brussels, Belgium); Medical University of Graz (Austria); KI Donatum, Karolinska Institute Stockholm, (Sweden); University of Lund (Sweden); institutions comprising the Utah Transplantation Affiliated Hospitals Cardiac Transplant Program (ie, University of Utah Health Science Center, Intermountain Medical Center and the Veterans Administration Salt Lake City Health Care System), Spectrum Health Universal Biorepository (Utah) and Gift of Life (Michigan). Ethical permission for these studies was granted by the regional ethics review board in Stockholm (DNR 2005/1029-31/2 and DNR 2010/313-31) and the Ethikkommission an der TU Dresden (BO-EK-50022020), and informed consent was provided by all individuals. Nondiseased human heart tissues were obtained by KI Donatum after informed consent from the individual or next of kin from the Swedish National Department of Forensic Medicine. Relevant clinical parameters are listed in Tables S1 and S2. Tissue from the left ventricle harvested at the time of heart transplantation, dissected, and stored at −80 °C until further processing. Tissue samples were processed in 3 ways: (1) nuclear isolation and fluorescence-activated cell sorting (FACS) sorting for ^14^C analysis, (2) paraffin embedding for subsequent histological analysis, or (3) isolation of intact cardiomyocytes from fixed tissue samples.

### Cardiomyocyte Nuclei Isolation

Large frozen heart pieces of the free ventricular wall (minimum, 10 g) were dissected and dissociated in a blender in lysis buffer (0.32 M sucrose, 10 mM Tris-HCl pH=8.0, 5 mM CaCl_2_, 5 mM MgAc, 2 mM EDTA) for 10 minutes. The tissue was further homogenized with a T-25 Ultra-Turrax probe homogenizer (IKA Germany) at 24 000 rpm for 10 s and afterward dounced with a type A pestle in a 40-mL glass douncer, applying 8 strokes. The nuclear isolate was filtered through 100- and 60-µm nylon mesh cell strainers and sedimented at 500× *g* for 10 minutes. The pellet was dissolved in sucrose buffer (2.0 M sucrose, 10 mM Tris-HCl, pH 8, 5 mM MgAc), and layered onto 10 mL of sucrose buffer in a BSA-coated centrifuge tube. The samples were spun down at 26 500× *g* for 60 minutes in a Beckman Avanti Centrifuge (Beckman Coulter). The nuclei pellets were dissolved in nucleus storage buffer (0.44 M sucrose, 10 mM Tris-HCl pH 8.0, 70 mM KCl, 10 mM MgCl_2_, 2 mM EDTA). All steps were performed at 4 °C.

### Immunolabeling and Flow Cytometry of Isolated Nuclei

A polyclonal rabbit anti-PCM1 ([pericentriolar material 1] Sigma Aldrich, HPA023374) antibody, conjugated to Alexa Fluor 488 or without conjugation to a fluorochrome, was added to the sample in a 1:800 dilution and incubated overnight. The next day, samples of isolated nuclei were filtered through 40 µm cell strainers (BD Bioscience) and labelled with DNA stain DRAQ5 (ThermoFisher, 62251, 1:1000) for ploidy analysis. Samples that were not processed with a primary antibody conjugated to a fluorochrome were incubated with a secondary anti-rabbit Alexa Flour 488 (1:1000) for 1 hour. Negative controls were incubated without primary antibody. Subsequently, the sample was loaded to the flow cytometer (FACS Aria II), single nuclei were identified by forward scatter height/forward scatter area gating, and cardiomyocyte nuclei were defined by gating the PCM1^+^ nuclei. After nuclei sorting, the populations were reanalyzed for purity. The sorted nuclei were spun down at 500× *g* for 10 minutes, dissolved in PBS, and kept at −20 °C until further processing. All steps were performed at 4 °C. The purity of the PCM1^+^ sorted fraction (n=52) was (median) 95.1% (interquartile range [IQR], 92.2% to 96.7%; Figure S2E). If the sorting purity was <100%, the DNA purity of the sample was calculated according to the following equation:


$$Purity_{x}=\frac{f_{x}*c_{x}}{f_{1}*c_{1}+f_{2}*c_{2}+...+f_{n}*c_{n}}$$

Where *x* is the sample being corrected, *f*_*x*_ is the nuclei purity measured in the FACS, *f*_1…*n*_ are the fractions of the *n* contaminating populations, *c*_1…*n*_ are the DNA contents of the contaminating nuclei (eg, 4n: *c*_1_=4), reflecting that polyploidy nuclei contain more DNA.

### DNA Extraction for ^14^C Birth Dating

DNA extraction from flow cytometry sorted PCM1^+^ nuclei of more than one million nuclei was performed under clean-room conditions; 500 µL of DNA lysis buffer (1% SDS, 5 mM EDTA-Na_2_, 10 mM Tris-HCl pH 8) and 6 µL of proteinase K (20 mg/mL; Life Technologies, EO0491) were added to each sample of sorted nuclei. The samples were incubated at 64 °C overnight. After adding half the volume of 5 M NaCl, the samples were vortexed for 15 seconds and spun down at 16 000× *g* for 3 minutes. The supernatants were transferred to 10-mL glass tubes, 3× volumes of 96% ethanol were added, and the glass tubes were inverted several times until a DNA precipitate became visible. The DNA pellet was washed in DNA washing buffer (70% ethanol, 0.5 M NaCl) 3 times for 15 minutes each. The washed DNA pellets were dissolved in 200 µL of sterile double distilled water (Gibco) and allowed to dry at 64 °C overnight. The dried DNA pellet was dissolved in 500 µL of sterile water overnight. The sample was analyzed for DNA concentration and purity by measuring the absorbance at 260 nm and 280 nm with a spectrophotometer (NanoDrop 1000, Thermo Scientific). Sample DNA abundance was median 42.9 µg DNA (IQR, 30.0–62.1 µg; n=52; Figure S2F).

### Accelerator Mass Spectrometry

Accelerator mass spectrometry measurements were performed blind to the identity of the samples and as described previously.^16^ Purified DNA samples (Figure S2F) were lyophilized to dryness. Excess copper (II) oxide was added to each dry sample, and the tubes were evacuated to 2.10^-3^ mbar and sealed with a high-temperature torch. The tubes were placed in a furnace set at 900 °C for 3.5 hours to combust all carbon to carbon dioxide. The carbon dioxide was then cryogenically purified, trapped, and reduced to graphite in the presence of an iron catalyst in individual reactors at 550 °C for 6 hours. Graphite targets were measured at the Department of Physics and Astronomy (Ion Physics, Uppsala University).^17^ Stable Isotope ratio, δ^13^C, was measured for each sample to correct for any isotopic fractionation. Strict laboratory practice was implemented to minimize stray background carbon contaminating the samples. Corrections for background carbon contamination introduced during sample preparation were made as described.^17^ ^14^C data are reported as decay-corrected Δ^14^C. The measurement accuracy was in the range of 1% to 3% (2σ).

### Histological Analysis of Cardiac Samples

Wheat germ agglutinin (WGA) staining was performed on 5-µm paraffin sections prepared from the formaldehyde fixed tissue samples. Sections were deparaffinized using xylene and ethanol and incubated with WGA, Alexa Fluor 488 Conjugate (1:1000; ThermoFisher, W11261, and diluted in PBS. Sections were washed 3 times with PBS and mounted with ProLong Gold Antifade Reagent containing DAPI (Life Technologies, P36935). Cardiomyocyte cross-sectional area was determined using CellProfiler 2.0^18^; analysis pipeline is provided as a data file (https://zenodo.org/record/7560219).

### Quantification of mtDNA Copy Number

To analyze mtDNA content total DNA was extracted from the tissue samples for processing with Takara Human mtDNA Monitoring Primer Set (category No.7246). Tissue biopsies were taken before the start of the nuclei isolation procedure, using a 6-mm wide disposable biopsy punch (Kai Medical, No. 48601) and homogenized by the Ultra-Turrax homogenizer at 24 000 rpm for 10 seconds in 1 mL of lysis buffer (0.32 M sucrose, 5 mM CaCl2, 5 mM magnesium acetate, 2 mM EDTA, and 10 mMTris-HCl, pH 8.0). DNA was isolated using 200 µL of the lysate, with the QIAamp DNA Blood Mini Kit (Qiagen, 51104) according to manufacturer instructions. DNA concentration and purity was determined using NanoDrop. Quantitative real-time PCR reactions were set up in a total volume of 25 μL using the Takara Human mtDNA Monitoring Primer Set (Takara, 7246) to quantify the relative number of copies of human mtDNA with respect to the nuclear genomic DNA (nDNA). Reactions were set up according to manufacturer protocol using 12.5 μL of SYBR Green master mix (Thermo Fisher, No. 4309155) and 10 ng of template DNA. Thermocycling was set up in the StepOne Real-Time PCR System (Applied Biosystems, No. 4376357).

### Tissue Digestion for Intact Cardiomyocyte Isolation

Tissue samples from the left ventricular wall and apical tissue obtained at the time of LVAD implantation were cut into 0.5- to 1-mm cubes and fixed in 3.7% formaldehyde solution for 100 minutes at room temperature. Subsequently, tissue blocks were washed 3 times with PBS for 30 minutes and then digested in a buffer consisting of collagenase B (Roche, 11088815001; 3.6 mg/mL) and collagenase D (Roche, 11088858001; 4.8 mg/mL) in PBS for 24 hours at 37 °C with gentle rotation. Isolated cardiomyocytes were gently centrifuged at 1× *g*, and the supernatant was removed before resuspending in PBS. For several samples, this procedure was repeated for an additional 24 hours on the remaining tissue.

### Immunostaining of Isolated Intact Cardiomyocytes

Isolated cardiomyocytes were allowed to settle for 15 minutes and were resuspended in 500 µL of blocking buffer with primary antibodies Mouse-α-Actinin,1:500 (Sigma Aldrich, A7811) and Rabbit-Connexin-43 (1:1000 [Sigma Aldrich, C6219]) for 30 minutes. Cardiomyocytes were washed once with PBS for 15 minutes and incubated with secondary antibody solution Alexa Fluor 555 anti-mouse, 1:1000 (Jackson Immuno Research, 711-546-152) and Alexa Fluor 488 anti-rabbit, 1:1000 (Abcam, ab150110) and Hoechst 33342 (1:1000 [ThermoFisher, 62249]) for 30 minutes. Cardiomyocytes were washed with PBS and resuspended in a small volume of PBS and pipetted onto a glass slide. Cardiomyocytes on the glass slide were allowed to settle and adhere before mounting (ProLong Gold antifade reagent; ThermoFisher, P36930). All imaging was performed on a Keyence BZ-X800E using 20× objectives with appropriate dichroic filters. Exposure times were chosen that allowed capturing of the full dynamic range without overexposure and were similar for all acquired images. At least 30 fields of view were recorded per sample in a nonoverlapping manner.

### Quantification of Ploidy and Binucleation

To identify nuclei in the intact cardiomyocytes isolated from fixed tissue and measure their integrated intensities, a CellProfiler 4.0^19^ pipeline was set up (https://zenodo.org/record/7560219). Nuclei identification was performed using the identify primary objects module with an adaptive thresholding method to account for background variances. The α-Actinin image was used to exclude noncardiomyocyte nuclei that do not fall within the α-Actinin positive areas. Further filtering by several neighbors and solidity was chosen to exclude large clumps of nuclear stains and irregular shapes, which indicated bad segmentation or accumulation of staining that did not resemble individual nuclei. Area and shape descriptors, as well as intensity descriptors, were exported for further analysis and integrated fluorescence intensity of the Hoechst stain was plotted as histograms to determine ploidy thresholds manually (Figure S2G through S2I). These thresholds were then used to overlay colored outlines on the overlay pictures for further analysis. Evaluation of the ploidy levels in mononucleated and binucleated cells was performed manually based on the overlay pictures, considering cardiomyocyte shape and border morphology using connexin-43 staining. Per sample, an average of 168 cardiomyocytes were analyzed with a minimum of 50.

### Echocardiography

Twenty-eight of 52 patients with advanced heart failure requiring circulatory support with continuous flow LVAD were prospectively enrolled at one of the institutions comprising the Utah Transplantation Affiliated Hospitals Cardiac Transplant Program (ie, University of Utah Health Science Center, Intermountain Medical Center, and Veterans Administration Salt Lake City Health Care System). Patients requiring LVAD support because of acute heart failure (eg, acute myocardial infarction, acute myocarditis, or postcardiotomy cardiogenic shock) were excluded. Serial echocardiographic assessments were performed prospectively within 1 month before LVAD implantation and at 1, 2, 3, 6, 9, and 12 months after LVAD implantation based on a previously described protocol.^20^ Patients were categorized as responders and nonresponders based on the absolute left ventricular ejection fraction (LVEF) change from pre–LVAD to post–LVAD implantation. Patients with an absolute LVEF increase ≥5% within 1 year on LVAD support were defined as responders.

### Differential Expression Analysis for DNA Repair Genes

We made use of a previously published single-cell sequencing data set of end-stage heart failure by Amrute et al.^21^ We downloaded the list of differentially expressed genes from Amrute et al (Table S7 by Amrute et al) for the comparison of “RRpost vs preLVAD HF.”^21^ These lists contain all differentially expressed genes (adjusted *P* value<0.05). Amrute et al used the DESeq2 package for this analysis, and the *P* values attained by the Wald test are corrected for multiple testing using the Benjamini and Hochberg method by default. In these lists, we then marked all genes that are members of the MSigDB v2023.2 Hallmark DNA repair gene set as DNA repair associated genes. Only 4 of the 150 Hallmark DNA repair genes were differentially expressed.

### Statistics

Values are given as mean±SD for normal distributed data and median±IQR for non-normal distributed data unless stated otherwise. Statistical tests were performed, and graphs were generated using Graphpad Prism Version 10 and SigmaPlot 13. One-way ANOVA was performed when the data had a normal distribution (Shapiro–Wilk) and the variances were equal (Brown–Forsythe). Kruskal–Wallis 1-way ANOVA on ranks was performed when any of the above conditions were not met. Appropriate post hoc multiple comparison tests were performed as indicated in the figure legends. *P*<0.05 was considered statistically significant. The Mann-Whitney rank sum test was used to compare 2 groups that were not normally distributed. Linear regression analysis was performed to calculate *R* and whether the slope is significant from 0. Compositional data underwent an isometric log-ratio transformation to address its compositional nature and facilitate subsequent statistical analysis.^22^ After the isometric log-ratio transformation, a principal component analysis was conducted to reduce the dimensionality of the data and identify patterns of variation among the groups. Only the first principal component was used for further analysis, reflecting the most significant axis of variation. The ANOVA was then employed to detect significant differences in first principal component scores across the 3 groups. To elucidate the specific differences identified by the ANOVA, a Tukey honestly significant difference post hoc test was performed, facilitating pairwise comparisons between the groups. The analysis was conducted using R 4.3.3 (R Core Team, 2024), leveraging the following packages: compositions for the isometric log-ratio transformation, multcomp for conducting Tukey honestly significant difference post hoc test, readxl for importing the data set, and tidyverse for data manipulation and visualization.

National Institutes of Health principles and guidelines for reporting preclinical research were followed. Experimenters were blind to group assignment and outcome assessment. For some analyses, a random subset of our patient population was selected (Table S3). No randomization was performed, as the groups were disease categories from human samples. Patient demographical details can be found in Tables S1 and S2. No sample size calculation was performed. Sample size was governed by tissue availability.

### Data Availability

All experimental data reported in this article will be shared by the lead contact upon request. All original code has been deposited at Zenodo and is publicly available (https://zenodo.org/record/7560219). Any additional information required to reanalyze the data reported in this article is available from the lead corresponding author upon request.

## RESULTS

### Cardiomyocyte Nuclear Ploidy and Multinucleation Increase in Cardiomyopathy

**Figure 1.**
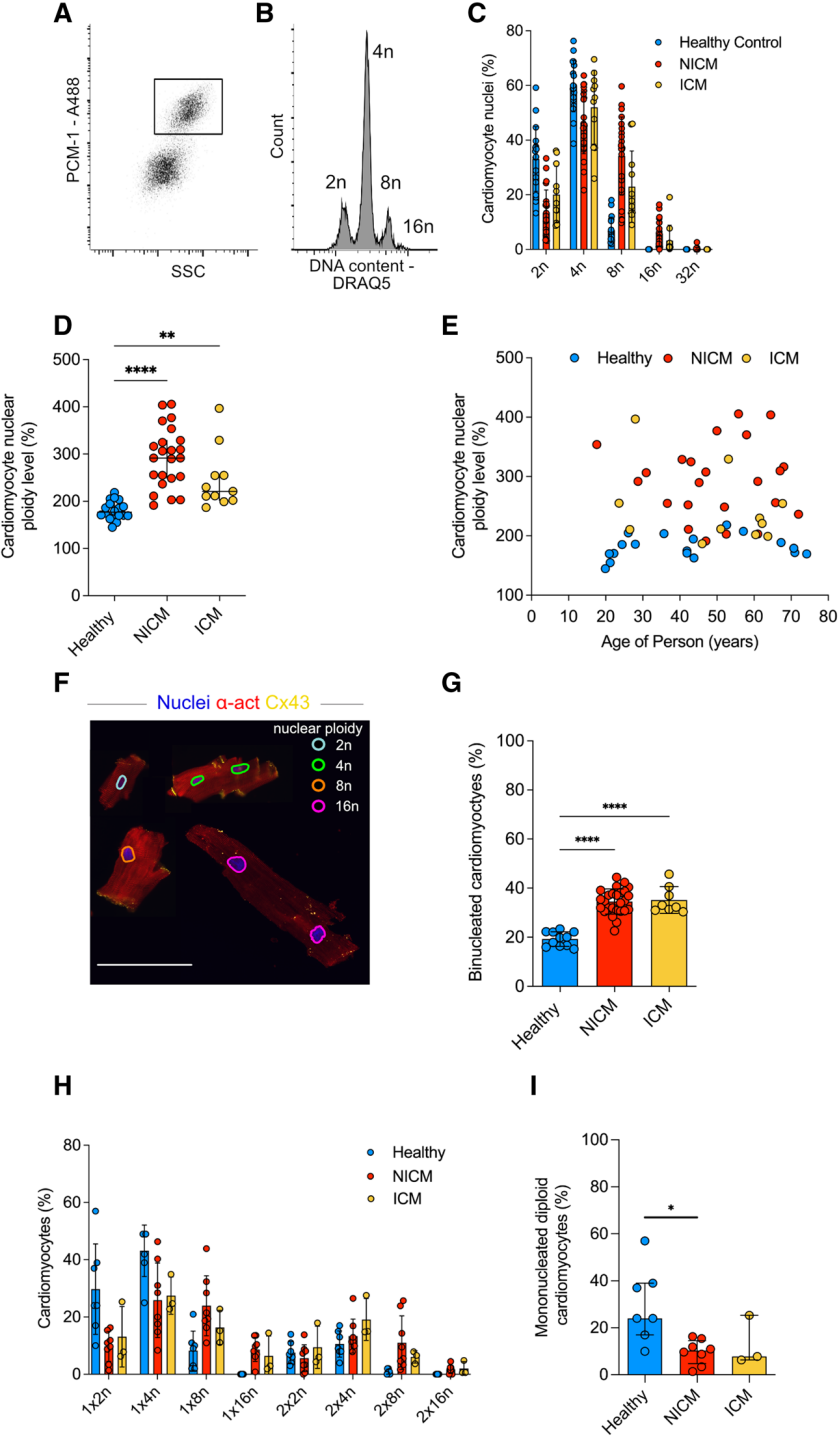
**Isolation of human cardiomyocytes and ploidy assessment. A**, Flow cytometry–based sorting enables identification and isolation of cardiomyocyte nuclei with antibodies against PCM1 (**inset**). **B**, Flow cytometry analysis of cardiomyocyte nuclei DNA content reveals their ploidy profile (shown is a representative NICM sample). **C**, The distribution of cardiomyocyte nuclear ploidy populations shows a shift to higher ploidy levels in NICM (n=23) and ICM patients (n=11) compared with healthy adults (n=11). **D**, Cardiomyocyte nuclear ploidy level determined by flow cytometry is greater in both NICM (n=23) and ICM patients (n=11) than in healthy adults ([n=11] eg, 100% corresponds to only diploid nuclei, 200% to only tetraploid nuclei; Kruskal–Wallis 1-way ANOVA on ranks, H=30.4; *P*<0.0001, post hoc Dunn **P=0.0063, ****P<0.0001), lines show median with interquartile range. **E**, Ploidy increase in cardiomyopathy is not related to the age of the adult patient (NICM, *R*=0.03, *P*=0.89; ICM, *R*=0.35, *P*=0.28). **F**, Image cytometry identifies cardiomyocyte ploidy classes. Cardiomyocytes were digested from tissue samples and stained for α-actinin (α-act) and connexin-43 (Cx43). The integrated intensity of the DNA dye was used to designate the nuclear ploidy class of each imaged nucleus, as shown by the multicolor overlay. Cx43 enabled us to determine whether cardiomyocytes were intactly dissociated at the intercalated discs. The figure shows a compilation of several cardiomyocytes of different ploidy and nucleation levels. Scale bar=100 µm. **G**, The percentage of binucleated cardiomyocytes determined by image cytometry is greater among NICM (n=26) and ICM hearts (n=8) compared with healthy hearts ([n=11] ordinary 1-way ANOVA, F=43.5; *P*<0.00001, post hoc Tukey; ****P<0.0001). **H**, Both binucleation and nuclear ploidy levels were determined in isolated cardiomyocytes from healthy (n=7) and pathologic hearts in samples from individual patients (NICM n=8; ICM n=3) with image cytometry. **I**, The percentage of mononucleated diploid cardiomyocytes is lesser among NICM (10.4%) and ICM (7.8%) compared with healthy hearts ([24.0%] Kruskal–Wallis 1-way ANOVA on ranks; H=7.7; *P*=0.01, *post hoc Dunn *P*=0.02). 2n indicates diploid; 4n, tetraploid; 8n, octaploid; 16n, hexadecaploid; ICM, ischemic cardiomyopathy; NICM, nonischemic cardiomyopathy; PCM1, pericentriolar material 1; and SSC, side scatter.

Cardiomyocytes often exit the cell cycle prematurely and become polyploid without forming daughter cells. We determined nuclear ploidy in cardiomyocytes of patients with failing hearts diagnosed with NICM and ICM (Table S1). Nuclei were isolated from tissue samples and cardiomyocyte nuclei identified using flow cytometry by their positive staining for PCM1 (mean±SD, 21.3±14.5% of all nuclei [Figure 1A; Figure S2]).^2,23,24^ Application of a DNA stain (DRAQ5) enabled the determination of their nuclear DNA content (Figure 1B). We found a shift toward higher nuclear ploidy levels in both NICM and ICM (Figure 1C) compared with healthy subjects (values taken from Bergmann et al^2^). As a measure of nuclear ploidy, we defined the degree of nuclear ploidy as a percentage in relation to 100% of a purely diploid (2n) reference sample.^2,3^

We observed a total increase in cardiomyocyte nuclear ploidy level from 176.9% (median [IQR, 169.8–196.9%]) in healthy hearts to 291.7% (250.4–341.4%) in NICM and 221.1% (202.1–255.0%) in ICM (Figure 1D). The increase in nuclear ploidy was not linked to the age of the patient (NICM, *R*=0.26, *P*=0.26; ICM, *R*=0.49, *P*=0.11; Figure 1E).

In a second approach, we isolated intact single cardiomyocytes and performed image cytometry to determine the number of nuclei per cardiomyocyte and their ploidy status (Figure 1F; Figure S2G and S2I). Using a sample set of healthy adults, we found 19.3±3.0% of cardiomyocytes to be binucleated (Figure 1G; Table S4), in agreement with previous studies, which also showed that this ratio remains unchanged in a healthy heart throughout the human lifespan.^2,25,26^ In contrast, we observed that cardiomyocytes isolated from NICM and ICM hearts showed significantly higher levels of binucleation compared with healthy hearts: 34.6±5.1% in NICM and 35.2±5.5% in ICM patients. Similarly, there was no correlation between the age of the patient and the ratio of binucleated cardiomyocytes (NICM, R=0.13, *P*=0.52; ICM, R=0.04, *P*=0.92; Figure S2D).

Furthermore, we determined individual ploidy levels within mononucleated and binucleated cardiomyocytes. This analysis revealed that the proportion of mononucleated diploid cardiomyocytes (1×2n) is smaller in NICM (median, 10.35% [IQR, 4.6–14.5%] and ICM (7.8% [6.3–25.3%]) compared with healthy hearts (24.0% [17.0–39.0%]; Figure 1H and 1I). Although both nuclear ploidy and multinucleation increase in NICM and ICM, the distribution of ploidy levels within the mononucleated and binucleated cardiomyocytes is independent of the number of nuclei per myocyte (Figure S2K). These measurements by image cytometry are consistent with the extent of nuclear ploidy determined by flow cytometry, shown by Bland–Altman comparison and linear regression analysis (Figure S2H through S2J). In conclusion, both demonstrate dramatically increased cardiomyocyte DNA content in diseased failing human hearts from both ICM and NICM patients.

### ^14^C Levels Establish Increased DNA Synthesis in Cardiomyocytes of Failing Hearts

**Figure 2.**
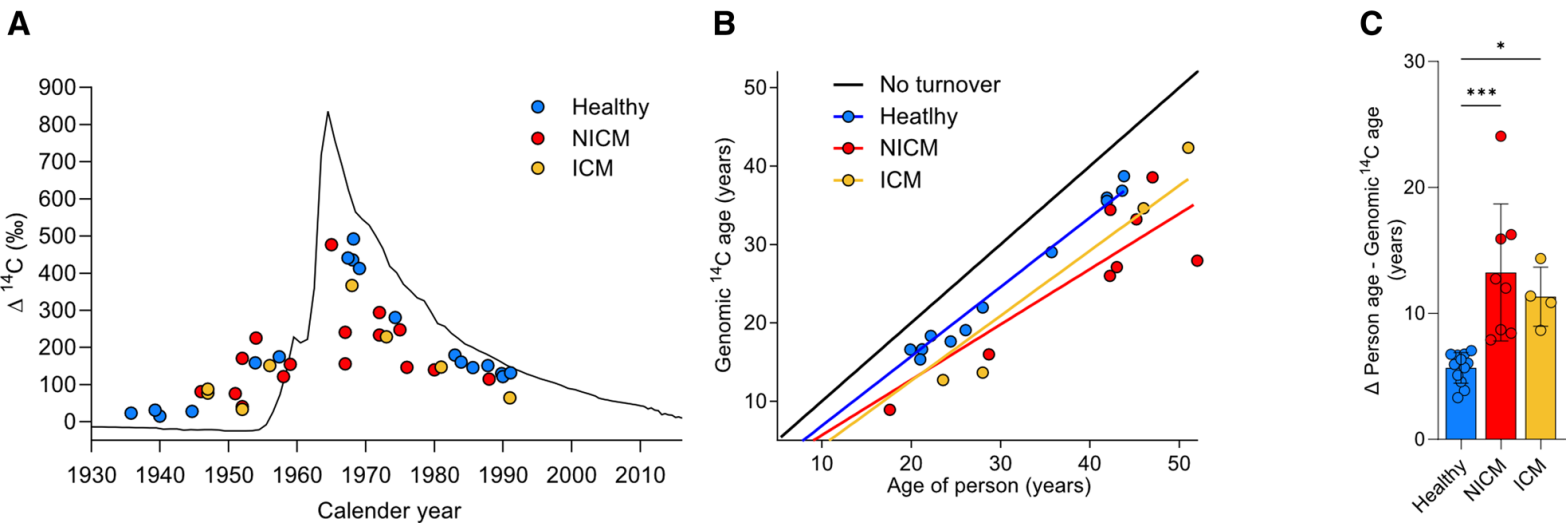
**^14^C levels indicate increased DNA synthesis in cardiomyocytes of failing hearts. A**, Presentation of ^14^C data. The black curve indicates the historic atmospheric ^14^C concentrations measured in the northern hemisphere.^43–45^ ^14^C measurements (delta ^14^C permille (‰) in relation to a universal ^14^C standard and corrected for radioactive decay,^46^ from heart samples are plotted as colored dots on the date of subject birth. Genomic ^14^C values of cardiomyocytes in healthy hearts ([blue dots] n=18; data from Bergmann et al, 2015) are close to atmospheric values at the time of birth, indicating a limited postnatal and adult renewal of cells. The deviation of ^14^C values of cardiomyocytes in nonischemic cardiomyopathy ([NICM] red dots; n=16) and ischemic cardiomyopathy (ICM) hearts (orange dots; n=8) from the atmospheric ^14^C curve suggest genomic DNA turnover. **B**, The estimated genomic ^14^C age of cardiomyocytes was calculated from subjects with postbomb birth dates and plotted at participant age. Black line indicates no turnover. Subjects born before the bomb spike (1963) were not included in this analysis because the genomic ^14^C age cannot be definitively determined, which related to shape of the atmospheric ^14^C curve.^43–45^ **C**, Both NICM (n=8) and ICM samples (n=4) show a greater deviation of the estimated genomic ^14^C age from the participant’s age compared with healthy hearts (n=12), suggesting increased DNA synthesis (Kruskal–Wallis 1-way-ANOVA on ranks, H=17.4; *P*=0.00017, post hoc Dunn multiple comparison, ***P=0.0004, *P=0.017).

We isolated cardiomyocyte nuclei (PCM1^+^) from NICM (n=16) and ICM patients (n=8), between 18 and 68 years of age (median, 51.5 years; Figure S2A) by FACS (Figure S2B and S2C) and determined the genomic ^14^C concentrations by accelerator mass spectrometry (Supplemental Methods, Tables S1 and S2; Figure 2A). The ^14^C amounts measured in NICM and ICM samples deviate more from the atmospheric ^14^C levels at the time of birth, than the values from healthy subjects,^2^ suggesting that there was more cardiomyocyte DNA synthesis in these patients. To better classify this divergence, we determined the average ^14^C age of the cardiomyocytes’ genomic DNA (Figure 2B; Figure S1A and S1B), which provides an initial estimation of cellular ages under the assumption that all cells were generated at the same time.^27^ In healthy subjects, the genomic ^14^C age of cardiomyocyte DNA was an average of 6 years (median [IQR, 4.7–6.7 years]) younger than the subject itself (Figure 2C; data set taken from reference 2). In contrast, we found the genomic ^14^C age of NICM cardiomyocytes to be 12.4 years (median [IQR, 8.5–16.1 years]), and of ICM cardiomyocytes to be 11.1 years (IQR, 9.2–13.6 years) younger than the individual, respectively. These observations demonstrate increased levels of DNA synthesis in cardiomyocytes from NICM and ICM patients compared with healthy controls.

### Reduced Cardiomyocyte Renewal in Heart Failure

We defined a population balance equation for ^14^C concentration structured cell populations, which we used to predict ^14^C concentration dynamics from cell renewal rates (see Mathematical Methods) using a Bayesian inference framework.^27^ The cell renewal rates are allowed to change in time, which enables us to model different rates before disease onset, during the disease, and after treatment with LVAD. For NICM and ICM patients, we assumed that polyploidization and multinucleation proceed physiologically until disease onset and increased thereafter until tissue procurement. For this assumption, we applied the pathological ploidy levels individually measured for all ^14^C dated samples (Figure 1D) and levels of binucleation determined for each population as depicted in Figure 1G.

**Figure 3.**
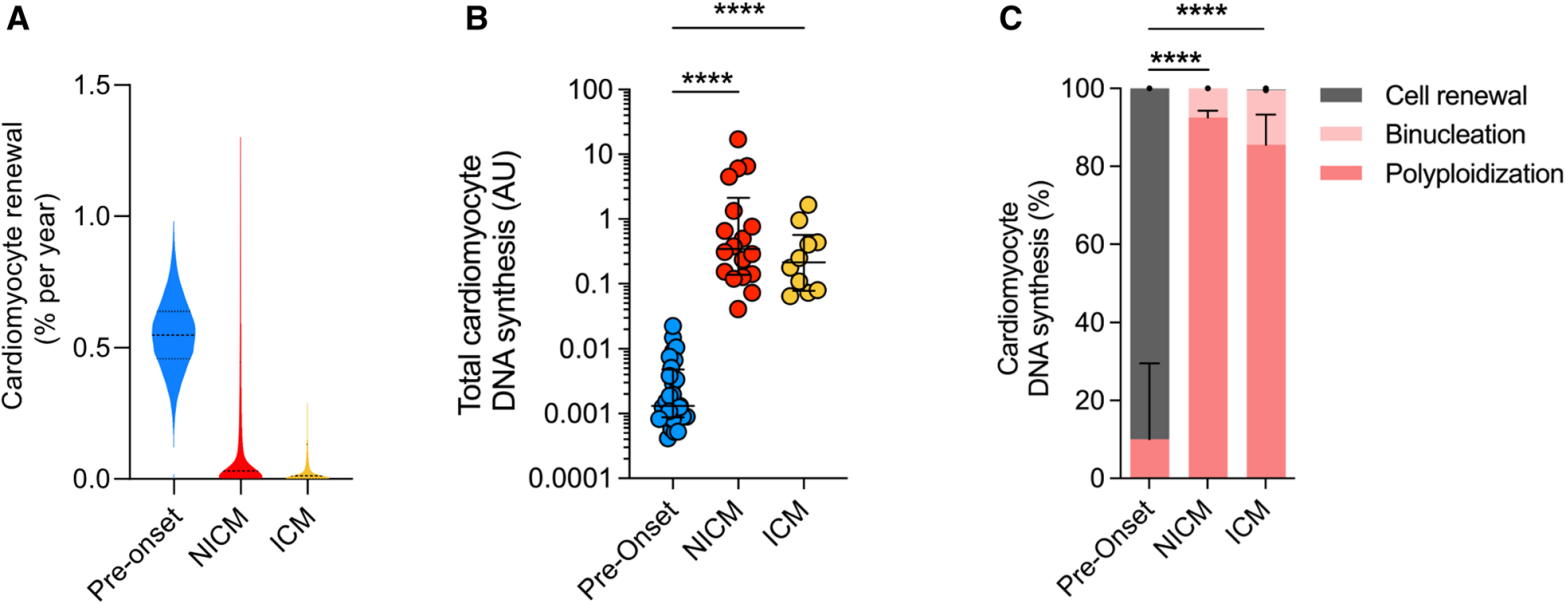
**Mathematic modelling of cardiomyocyte renewal dynamics in failing hearts. A**, Violin plot of the probability distribution of the true parameter of the turnover rate before onset and after onset for nonischemic cardiomyopathy (NICM) and ischemic cardiomyopathy ([ICM] Monte Carlo samples, n=200 000; Supplemental Material). Dashed line indicates the median turnover rate; dotted lines indicate the range of 50% credibility interval (ie, true value is in this range with 50% probability). In the disease phase, the percentage of newly formed cardiomyocytes drops from 0.6% ([0.5–0.6%] median [interquartile range]) to 0.03% (0.002%–0.5%) in NICM, and 0.01% (0.001%–0.1%) in ICM. **B**, The annual amount of DNA synthesis predicted by our model using the fitted renewal rate and the case-specific data such as age, date of diagnosis, and polyploidization. It shows an increase in both NICM and ICM compared with healthy hearts (Kruskal–Wallis 1-way ANOVA on ranks, H=41.6; *P*<0.00001, post hoc Dunn multiple comparison; ****P<0.0001). **C**, Origin of the annual amount of synthesized DNA attributed to cell renewal, binucleation and nuclear polyploidization estimated in diseased hearts predicted by our model (Supplemental Methods). After disease onset in NICM and ICM patients <0.3% of DNA synthesis can be attributed to proliferation-based cell renewal. The compositional data were analyzed as described in the statistics section. ANOVA revealed significant effects of condition on the source of newly formed DNA (*F*(2, 53)=1424, *P*<2e-16), post hoc Tukey honestly significant difference test; ****P<0.00001).

To establish the rate of cardiomyocyte renewal occurring specifically during the disease phase, we defined 2 phases of cardiomyocyte renewal: before and after the disease onset (first occurrence of heart failure symptoms). In this 2-phase model, we assumed that the renewal rates before disease onset are the same as in healthy subjects (0.55% per year). The fitted renewal rates after disease onset resulted in much lower median renewal rate values of 0.03% per year for NICM patients and 0.01% per year for ICM patients, corresponding to an 18- and 50-fold reduction, respectively, compared with the healthy heart (Figure 3A).

To test for the possibility that changes in cardiomyocyte renewal could be part of disease ontogeny in NICM patients, we used our simplified model assuming a constant lifelong cardiomyocyte renewal (Supplemental Methods). We find that the distribution of cardiomyocyte renewal is wider with a similar median in NICM compared with healthy hearts (0.55% per year in healthy hearts and 0.59% per year in NICM), suggesting that there is no overall early loss of the capacity to renew in the NICM group (Supplemental Methods).

The fact that cardiomyocyte renewal is greatly reduced in heart failure suggests that the increased cardiomyocyte DNA synthesis, as determined by changes in genomic ^14^C levels in failing hearts, is almost exclusively related to polyploidization and multinucleation. (Figure 2B and 2C). To quantify this, we inferred total DNA synthesis from genomic ^14^C concentration in cardiomyocytes and examined the contribution of polyploidization, multinucleation, and cardiomyocyte renewal attributable to proliferation to total DNA synthesis. Applying our 2-phase mathematical model, we found that most of the DNA synthesis at the timepoint 1 year before disease onset was associated with proliferation-based cardiomyocyte renewal (median, 90.0% [IQR, 70.5–93.0%]; Figure 3C), whereas this rate dropped dramatically to values of 0.2% (median [IQR, 0.1–0.4%] and 0.2% (median [IQR, 0.1–0.7%]) at the timepoint of collection during the disease phase of NICM and ICM hearts, respectively. In those samples, most of the DNA synthesis was indeed associated with polyploidization (NICM median, 92.5% [IQR, 89.8–94.3%]; ICM 85.5% [80.5–93.3%]) and multinucleation (NICM, 7.5% [5.0–7.5%]; ICM, 14.0% [6.0–19.5%]; Figure 3C).

In summary, polyploidization and multinucleation explain >99.5% of the ^14^C incorporated into the DNA of failing cardiomyocytes, whereas cardiomyocyte renewal drops drastically to minimal levels.

### Mechanical Unloading and Circulatory Support Increase Cardiomyocyte Renewal in Patients With LVAD-Mediated Improvement of Cardiac Structure and Function

Having observed the dramatically reduced cardiomyocyte renewal rate of the failing heart, we inquired whether this could be rescued by mechanical unloading and circulatory support. We analyzed advanced heart failure patients (including NICM and ICM) receiving LVAD therapy for 3 to 43 months (Figure S4A and S4B). Failing hearts not supported with an LVAD were used as the reference group (non-LVAD). It has been shown that in advanced heart failure patients, LVAD mechanical unloading and circulatory support can improve cardiac function to varying degrees.^8,20^ Based on these LVAD studies as well as investigations of other heart failure therapies,^28,29^ we categorized all patients with an absolute increase of LVEF >5% as responders. To compare cardiomyocyte hypertrophy, we measured their cross-sectional area in these subgroups (Figure S4C). Responders showed smaller cardiomyocyte cross-sectional areas (832.8±307 µm^2^) than patients with no improvement in LVEF ([nonresponders] 1123±322 µm^2^; Figure S4D). Moreover, we studied mitochondrial abundance quantified by the relative copy number of human mitochondrial DNA by real-time PCR and normalized to genomic DNA copy numbers. Normalized mitochondrial DNA was lower in LVAD responders (median, 1984 [IQR, 1143–3736]) than in the LVAD nonresponder group (4025 [2918–5233]; *P*=0.02]) and non-LVAD patients (3487 [2821–5962]; *P*=0.01]; Figure S4E). Reversal of cardiomyocyte hypertrophy and decrease in mitochondrial load are 2 parameters of reverse remodeling that we have previously reported to be associated with mechanical unloading and circulatory support.^30^

In contrast to previously published work,^13,31^ we did not detect changes in nuclear ploidy or multinucleation in mechanically unloaded hearts using flow cytometry and image cytometry (Figure S4F and S4G). Neither responders nor nonresponders showed significant differences in these parameters compared with non-LVAD advanced heart failure patients.

**Figure 4.**
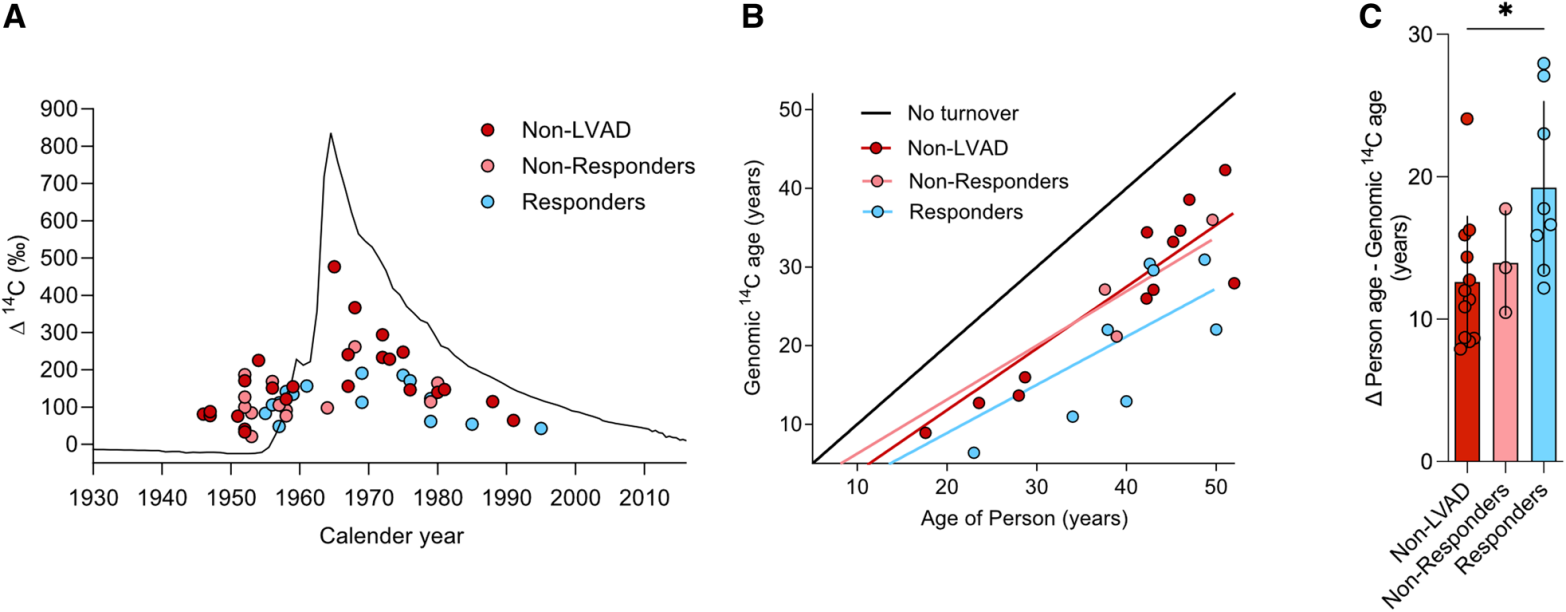
**^14^C levels in cardiomyocytes of LVAD-supported hearts. A**, Presentation of ^14^C data from unloaded hearts. ^14^C measurements from heart samples are plotted as colored dots on the participant’s date of birth for diseased non-LVAD hearts ([red dots; n=24] these correspond to nonischemic cardiomyopathy [NICM] and ischemic cardiomyopathy [ICM] samples as shown in Figure 2A), LVAD nonresponders (n=13) and LVAD responders (n=15). The location of the dots from responder subjects suggests an increased deviation from the atmospheric ^14^C curve compared with non-LVAD subjects. **B**, The estimated genomic ^14^C age of cardiomyocytes was calculated from subjects with post-bomb birth dates and plotted at the person’s age. The black line indicates no turnover. **C**, Responders (n=8) show genomic ^14^C age, which is 19.3 years younger than the participant on average, compared with 13.9 years for LVAD nonresponders (n=3) and 12.6 years for non-LVAD patients (n=12). Ordinary 1-way ANOVA, *F*=4.2; *P*=0.03, post hoc Tukey, *P=0.025. LVAD indicates left ventricular assist device.

Next, we determined the ^14^C concentration in cardiomyocyte DNA in LVAD responders (n=15) and nonresponders ([n=13] see Methods; Tables S1 and S2) to establish their ages and renewal rates compared with non-LVAD patients ([n=24] Figure 4A). ^14^C concentrations of responders deviate more from atmospheric levels than non-LVAD patients. To classify potential fundamental differences in cardiomyocyte age within the three subgroups, we determined the genomic ^14^C ages as a first estimate and plotted these against the age of the patient (Figure 4B). The group of responders was shown to differ the most from the no turnover prediction line. We compared these average genomic ^14^C DNA ages with the ages of the subjects and observed that it was 12.6±4.6 years fewer in the non-LVAD patients and 13.9±3.6 years for nonresponders, compared with 19.3±6.0 years in responders (Figure 4C).

**Figure 5.**
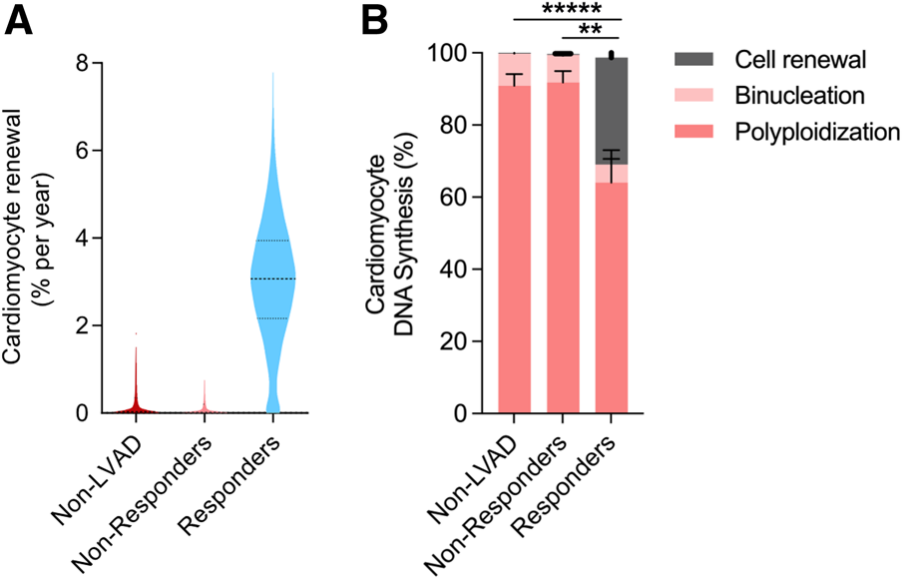
**Mechanical unloading increases cardiomyocyte renewal in LVAD responders. A**, Cardiomyocyte renewal rate as determined using the 2-phase renewal model. In cardiomyopathy, the percentage of newly formed cardiomyocytes per year is 3.1% (2.2–3.9; median [interquartile range]) in LVAD responders, compared with 0.03% (0.002%–0.4%) in the non-LVAD group and 0.02% (0.001%–0.2%) in LVAD nonresponders. **B**, Percentage of DNA synthesis attributed to cell renewal, binucleation, and nuclear polyploidization determined at the time of sample collection. The compositional data were analyzed as described in the statistics section. The compositional data were analyzed as described in the statistics section. ANOVA revealed significant effects of condition on the source of newly formed DNA (*F*[2, 49]=21.05; *P*<3e^-07^); post hoc Tukey honestly significant difference test, *****P<1e^-7^, **P<0.001. LVAD indicates left ventricular assist device.

To further assess the impact of LVAD treatment on cardiomyocyte renewal during the disease process, we applied the aforementioned 2-phase model of cardiomyocyte renewal. The fitted renewal rates after disease onset resulted in median renewal values of 0.03% per year for non-LVAD and 0.02% for LVAD nonresponder patients; in contrast, renewal values in LVAD responders were 3.1% per year (Figure 5A). In responders, 29.7% (median [IQR, 8.5–54.5%]) of all cardiomyocyte DNA synthesis can be attributed to cell renewal, contrasting with only 0.26% (0.1–0.6%) and 0.23% (0.01–1.0%) in nonresponders and non-LVAD patients, respectively; almost all DNA synthesis is attributed to polyploidization and binucleation (Figure 5B). This suggests a fundamental difference in the regenerative capacity in patients with advanced heart failure exhibiting cardiac functional improvement during LVAD mechanical unloading.

## DISCUSSION

We have used retrospective ^14^C birth dating to investigate cardiomyocyte renewal in cardiac tissue samples from patients with advanced heart failure. We report a major increase in DNA synthesis in all patient groups, leading mainly to cardiomyocyte polyploidization and binucleation. In patients without LVAD-mediated mechanical unloading, we found evidence of severely reduced cardiomyocyte generation compared with healthy subjects. In contrast, mechanically unloaded hearts exhibiting cardiac functional improvement (responders) showed a marked increase in cardiomyocyte renewal to levels approximately 6-fold higher than in healthy subjects. This demonstrates that there is a strong regenerative potential in the myocardium of a group of patients with advanced heart failure.

It is important to consider the stability of the DNA for the validity of the current approach, as nucleotide exchange in the absence of cardiomyocyte renewal would give a false impression of a renewing cardiomyocyte cell population.^32^ Most DNA repair is linked to cell cycle activity,^33,34^ and therefore does not change the ^14^C concentrations in cardiomyocytes undergoing ploidy and cell renewal. Previously, we were not able to detect DNA repair or other DNA alterations in a nondividing cell population of human cortical neurons from healthy and stroke patients by ^14^C birth dating,^35,36^ which indicates that even after a stroke during which there is massive DNA damage and repair, the impact on the ^14^C concentration in genomic DNA is below the level of detection. We report cardiomyocyte renewal <0.03% in failing hearts, which would be even less with DNA repair being detected by ^14^C birth dating but it is not accounted for. Moreover, there is no evidence that DNA damage is increased in unloaded hearts,^36^ which could lead to an overestimation of cellular renewal in LVAD responders. We analyzed published single-cell RNA sequencing data of cardiomyocytes in failing hearts and LVAD responders’ hearts,^21^ demonstrating that most DNA repair genes were not differently regulated (146 out of 150), supporting again the validity of our findings (see Methods).

Our model predicts minimal cardiomyocyte renewal rates in both NICM and ICM patients (Figure 3A), suggesting that cardiomyocyte renewal dynamics in failing hearts are not necessarily dependent on the underlying heart disease etiology. Even in patients with a 10-year history of cardiomyopathy, <0.2% of the existing cardiomyocytes would have been exchanged, which is much lower than in the healthy heart and unlikely to contribute to a clinical regenerative effect. The reduced capacity of cardiomyocytes to self-renew could even contribute to further disease progression.

Most studies on heart regeneration in both neonatal and adult rodents found an increase in cardiomyocyte proliferation directly after a myocardial lesion.^37,38^ Newly formed cardiomyocytes generated mainly in the acute phase of the disease would have been detected with ^14^C birth dating unless these cells selectively died before ^14^C measurement. Thus, if these cardiomyocytes existed, they would be short-lived and have had a limited effect on regeneration. Because of the chronic nature of heart failure, the minimal renewal rate is not surprising and could be a consequence of the hostile microenvironment present in failing hearts, which stimulates cell cycle activity but fails to accommodate successful cytokinesis.^39^

Mechanical unloading and circulatory support have been shown to mediate structural, cellular, and molecular changes in the failing myocardium of advanced heart failure patients with different degrees of functional cardiac improvement, which is referred to as reverse remodeling.^8,20^ Those patients showed an increase in cardiomyocyte renewal rate to 3.1% per year (Figure 5A), which is approximately 6-fold higher than physiological cardiomyocyte renewal levels,^2^ along with reduced cardiomyocyte hypertrophy, and a lower mitochondrial load in these patients (Figure S4D and S4E).^7,30^ Those features of reverse remodeling have previously been associated with markers of cardiomyocyte proliferation; however, this does not show whether new cells were generated and survived long-term, which we demonstrate herein.^14^ Because of the nature of our study, it is difficult to understand the exact causal relationship between reverse remodeling, functional improvement and cardiomyocyte renewal in the patients studied. However, our results demonstrate that reverse remodeling likely plays an important role in the observed phenotype.

The discovery of a latent cardiomyocyte regenerative potential in the adult human heart identifies an attractive target for new therapies. This motivates studies to unveil the molecular regulation of this process, which would facilitate the development of pharmaceutical therapies for heart regeneration. For instance, mechanical unloading might reverse metabolic cascades that increase reactive oxygen species production. This, in turn, can reduce oxidative DNA damage and activation of the DNA damage response pathway^40^ that causes cell cycle arrest in cardiomyocytes.^41^ Indeed, we found a reduction of endoreplication and higher rates of cardiomyocyte proliferation in responders (Figure 5B). This suggests that a successful approach for cell replacement strategies could be to selectively stimulate cytokinesis in already cycling cardiomyocytes.^42^

In summary, our findings reveal a strong cardiomyocyte regenerative potential in advanced heart failure patients, which motivates studies focusing on the molecular mechanisms implicated in myocardial recovery and the discovery of pharmaceutical strategies to promote it.

## ARTICLE INFORMATION

### Acknowledgments

The authors thank M. Toro and S. Giatrellis for assistance and advice in flow cytometry, K. Håkansson for accelerator mass spectrometry sample preparation, G. Possnert for help with analysis of the accelerator mass spectrometry samples, A. Felker and G. Eppens for help with flow cytometry sorting and DNA purification, and R. Savill for polyploidy analysis of isolated cardiomyocytes.

### Sources of Funding

This work was supported by the Flow Cytometry Facility, the Light Microscopy Facility, and the Histology Facility, all core facilities of the CMCB Technology Platform at TU Dresden. O.B. was supported by the Center for Regenerative Therapies Dresden, the Karolinska Institutet, the Swedish Research Council, the Ragnar Söderberg Foundation, the Åke Wiberg Foundation, and the LeDucq foundation. J.F. was supported by the Swedish Research council, the Swedish Cancer Foundation and Knut och Alice Wallenbergs Stiftelse. L.B. acknowledges support by the BMBF (grant No. 031L0293D). The model simulations and Bayesian inference were performed on HPC resources granted by the ZIH at TU Dresden. S.D. was supported by American Heart Association (grant No. 16SFRN29020000), National Heart Lung and Blood Institute (grant Nos. R01HL135121, R01HL132067, and R01HL166513), US Department of Veterans Affairs (merit review award Nos. I01CX002291 and I01BX006306), and the Nora Eccles Treadwell Foundation. C.P.K. was supported by National Heart Lung and Blood Institutes (grant No. 2T32HL007576-36).

### Disclosures

None.

### Supplemental Material

Supplementary Methods

Figures S1–S4

Tables S1–S4
